# Notch Signaling in Endothelial Cells: Is It the Therapeutic Target for Vascular Neointimal Hyperplasia?

**DOI:** 10.3390/ijms18081615

**Published:** 2017-07-25

**Authors:** Ding-Yuan Tian, Xu-Rui Jin, Xi Zeng, Yun Wang

**Affiliations:** 1 Trainee Brigade, Third Military Medical University, Chongqing 400038, China; tiandy75@outlook.com (D.-Y.T.); jimtmmu@163.com (X.-R.J.); 2 Department of Cell Biology, Third Military Medical University, Chongqing 400038, China; zency_xi@163.com

**Keywords:** neointimal hyperplasia, vascular injury, endothelial cell, Notch signaling, biological function

## Abstract

Blood vessels respond to injury through a healing process that includes neointimal hyperplasia. The vascular endothelium is a monolayer of cells that separates the outer vascular wall from the inner circulating blood. The disruption and exposure of endothelial cells (ECs) to subintimal components initiate the neointimal formation. ECs not only act as a highly selective barrier to prevent early pathological changes of neointimal hyperplasia, but also synthesize and release molecules to maintain vascular homeostasis. After vascular injury, ECs exhibit varied responses, including proliferation, regeneration, apoptosis, phenotypic switching, interacting with other cells by direct contact or secreted molecules and the change of barrier function. This brief review presents the functional role of the evolutionarily-conserved Notch pathway in neointimal hyperplasia, notably by regulating endothelial cell functions (proliferation, regeneration, apoptosis, differentiation, cell-cell interaction). Understanding endothelial cell biology should help us define methods to prompt cell proliferation, prevent cell apoptosis and dysfunction, block neointimal hyperplasia and vessel narrowing.

## 1. Introduction

Neointimal hyperplasia is an exaggerated wound-healing process that occurs in the vessel wall after injury. As a major morphological feature of many cardiovascular diseases (CVD), such as atherosclerosis and hypertension, neointimal hyperplasia is also responsible for the stenosis of vascular surgery, including bypass grafting, angioplasty and arteriovenous fistula [1,2]. The development of neointimal hyperplasia is a complex process initiated by the damage of endothelial cells (ECs) and exposure of vascular smooth muscle cells (VSMCs) to circulating blood elements. The process is further characterized by proliferative and inflammatory responses including VSMC proliferation and migration, platelet aggregation, leukocyte recruitment and extracellular matrix (ECM) deposition. Finally, EC proliferation or regeneration occurs at the lesion [3].

One of the important candidates for triggering neointimal formation is the dysfunction of endothelium. In the cardiovascular system, the endothelium is not only a barrier between the circulating blood and VSMCs, but also, it releases factors that regulate vascular tone, vessel growth, platelet function and coagulation [4]. For the underlying VSMCs, ECs could harmonize their growth and regression, through direct contact with VSMCs or secreted mediators that affect their proliferation, migration and death. In addition, ECs can regulate the thickness of intimal ECM through secreting enzymes, or inhibitors of these enzymes, which are able to degrade its components. The balance of these endothelial-derived activities regulates vessel development and vascular remodeling [5].

Recent advances in the understanding of the biology of neointimal formation indicate that ECs play a central role in the development of intimal hyperplasia during the process of vascular reconstruction. However, the mechanism of vascular neointimal hyperplasia is complicated, and a number of different intercellular signaling pathways has been implicated in this process. These pathways include the vascular endothelial growth factor (VEGF) pathway, the transforming growth factor-β (TGF-β) pathway, the Notch pathway, the Wnt pathway and many other pathways [6,7,8]. Among these pathways, the evolutionarily-conserved Notch signaling pathway controls cell fate through local cell-cell interactions. It plays a key role in the development of the cardiovascular system, as well as in the stability and remodeling of the vessel wall [9,10]. The purpose of this review is to summarize certain aspects of Notch signaling in endothelial cell biology and suggest how this knowledge might be used to reduce neointimal hyperplasia in cardiovascular disease and vascular surgical procedures.

## 2. The Notch Signaling Pathway

**Figure 1:**
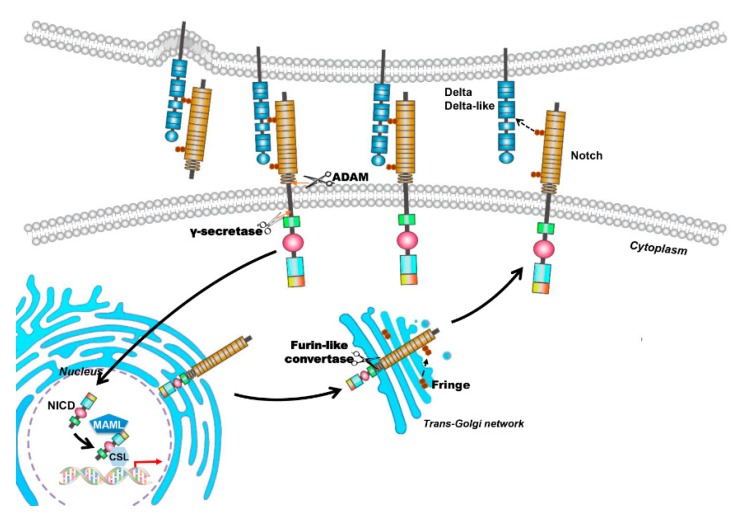
The canonical Notch signaling pathway. Mammal Notch family members are composed of four Notch transmembrane receptors (Notch-1, Notch-2, Notch-3 and Notch-4) and five typical transmembrane ligands (Delta-like 1, Delta-like 3 and Delta-like 4, Jagged-1 and Jagged-2). Notch receptors are synthesized as single-chain precursors and transported to the Golgi apparatus (Black arrow points to the Golgi apparatus). In the Golgi apparatus, the precursors are cleaved into an extracellular and a transmembrane subunit by furin and modified by glycosyltransferases Fringe. Then the matured proteins are transported and inserted into cell membrane (Black arrow points to the cell membrane). Interaction between Notch receptors and their ligands triggers the canonical Notch signaling pathway. The transmembrane Notch receptor is cleaved by a disintegrin and metalloproteinases (ADAM) to remove the extracellular subunit, and then, a multisubunit membrane protease γ-secretase catalyzes the second proteolytic cleavage that gives rise to the translocation of the Notch intracellular domain (NICD) into the nucleus (Black arrow points to the nucleus). In the nucleus, NICD binds with a transcription factor (Black arrow in the nucleus), RBP-Jκ (also known as CSL for CBF1/Su(H)/Lag-1), coactivator Mastermind-like (MAML) proteins, and forms an activated transcriptional complex. Then, the activated complex upregulates the expression of target genes (red arrow), such as hairy and enhancer of split (HES)-1, -5, -7 and HES-related repressor protein (HERP)-1 to -3. The deep blue pentagon represents coactivator MAML protein and the light blue hexagon represents transcriptional factor CSL. The orange arrows indicate cleavage sites; the arrow with dotted line in Golgi aparatus indicates protein glycosylation by Fringe and the arrow with dotted line between Notch ligand and receptor indicates the interaction of the two proteins.

Notch signaling is significant in determining cell fate and regulating cell proliferation, apoptosis and differentiation [11,12]. It was originally identified in *Drosophila*, in which a mutant allele gives rise to a notched wing [13]. Mammals express four Notch transmembrane receptors (Notch-1, Notch-2, Notch-3 and Notch-4) and five typical transmembrane ligands (Delta-like 1 (Dll-1), Delta-like 3 (Dll-3) and Delta-like 4 (Dll-4), Jagged-1 and Jagged-2). Notch receptors are synthesized as single-chain precursors and cleaved into an extracellular and a transmembrane subunit by furin like convertase in the Golgi apparatus (Figure 1). These two subunits are held together on cell membrane by non-covalent bonds. Interaction of Notch receptors with their ligands leads to the transmembrane Notch receptor cleaved by a disintegrin and metalloproteinases (ADAM) proteases to remove the extracellular subunit. After that, a multisubunit membrane protease γ-secretase is responsible for the second proteolytic event that gives rise to the translocation of the Notch intracellular domain (NICD) into the nucleus. In the nucleus, NICD binds with a transcription factor, RBP-Jκ (also known as CSL for CBF1/Su(H)/Lag-1), and forms an activated transcriptional complex. Then, the activated complex upregulates the expression of target genes, such as hairy and enhancer of split (HES)-1, -5, -7 and HES-related repressor protein (HERP)-1 to -3 [14].

The fact that Notch signaling plays a crucial role in vascular biology has been clearly demonstrated. Abnormalities in vascular system caused by mutations of Notch receptors (Notch-1, -2, -4), ligands (Dll-1, -3, Jagged-1, -2) and effectors (HES-1, -5, -7, HERP-1) in mice have been reviewed in detail [15]. The disruption of Dll-4 or RBP-Jκ in mice also results in lethality due to defects in vascular remodeling or angiogenesis [16]. Human hereditary vascular disorders, such as cerebral autosomal-dominant arteriopathy with subcortical infarcts and leukoencephalopathy (CADASIL) and Alagille syndrome (AGS), which manifest abnormalities in the cardiovascular system, are caused by mutations of Notch-3 and Jagged-1, respectively [17,18].

Recent studies support the emerging concept that Notch signaling is also involved in the development of neointimal hyperplasia. The increased Notch-1 signaling mediates neointimal formation in integrin β3(−/−)-induced arteriovenous graft occlusion through impairing EC regeneration [19]. Dll-4-mediated Notch activation promotes VSMC proliferation and migration in vein graft lesions and leads to vein graft failure [20,21]. Moreover, blocking the Notch pathway by using soluble Jagged-1 or by genetic deletion of the RBP-Jκ gene can inhibit neointimal formation after vessel injury [22,23].

As mentioned above, the triggering event in neointimal hyperplasia is EC damage. The ligands, receptors and other components of Notch signaling are expressed in ECs of different vascular origins [15,24]. During vascular injury, the Notch signal is definitely modulated, resulting in EC proliferation, apoptosis and differentiation. How these physiological alterations and barrier function impairments of ECs contribute to neointimal formation will be discussed later.

## 3. Endothelial Cell Proliferation and Regeneration

Notch signaling is an important regulator of EC proliferation and regeneration. During angiogenesis, Notch signaling suppresses EC proliferation and acts as an angiogenic “off” switch by making ECs unresponsive to VEGF [25,26]. It is estimated that only 0.01% of cells are actively proliferating in the vasculature of the adult [27,28]. Notch activation seems absent in vessels when ECs are proliferating at the early stages of angiogenesis; however, Notch is reactivated when ECs stop proliferating and vessels begin to stabilize [29,30]. Activation of Notch-1 and Notch-4 by Jagged-1 or Dll-4 reduces EC proliferation and contributes to contact inhibition of ECs [24,31]. Consistently, an extensive literature also reported that genetic or shRNA-mediated Dll-4 blockade in ECs leads to increased proliferation [32].

**Figure 2:**
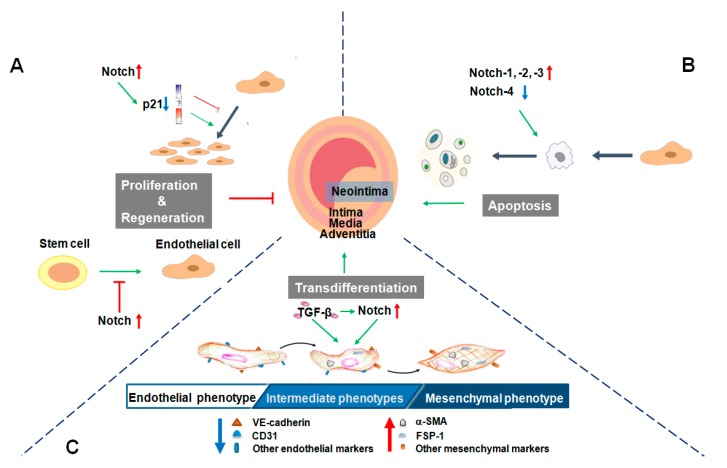
Notch signaling modulates endothelial cells’ (EC) fate associated with neointimal hyperplasia. (**A**) EC proliferation reduces neointimal hyperplasia. Notch signaling may have dual function in EC proliferation, which is dependent on the relative level of p21 expression. If the expression of p21 is above the level, Notch activation inhibits EC proliferation, however, ECs grow and proliferate when the expression of p21 is below the level. In addition, activation of Notch blocks EC regeneration and induces neointimal hyperplasia; (**B**) EC apoptosis contributes to neointimal hyperplasia. Upregulation of Notch-1, Notch-2, Notch-3 and downregulation of Notch-4, promote EC apoptosis, which further promotes neointimal hyperplasia; (**C**) Endothelial-mesenchymal transition (EndMT) contributes to neointimal hyperplasia. Notch activation can trigger EndMT and promote neointimal hyperplasia. The canonical EndMT inducer TGF-β (transforming growth factor-β) also can activate the Notch signaling pathway. FSP-1, fibroblast-specific protein 1; CD31, also known as platelet endothelial cell adhesion molecule-1 (PECAM-1).

Compared with its role in angiogenesis, the role of Notch signaling during neointimal formation is more complicated. As shown in Figure 2, Notch activation suppresses EC proliferation, regeneration and promotes neointimal formation (Figure 2A). In integrin β3 knockout mice, the increased Notch-1 signaling inhibits circulating angiogenic cells’ (CACs) homing and differentiation, delays endothelial regeneration and promotes neointimal formation at the sites of arteriovenous grafts [19]. It is also reported that endothelial progenitor cells’ (EPCs) activity is greater in Notch-1(+/−) EPCs than in wild type (WT) EPCs, and subsequently, transplantation of Notch-1(+/−) bone marrow accelerates endothelial recovery after arterial injury in WT mice. Consistently, inhibition of Notch-1 mRNA expression in EPCs by cholesterol enhances EPCs’ activity and accelerates EC regeneration after arterial injury in atherosclerosis mice [33].

One of the characteristics of pulmonary hypertension is vascular remodeling and vascular neointimal thickening. Notch-1 and Notch-4 are detected in rat pulmonary artery ECs. Following the pulmonary hypertension induction, mRNA expression levels of Notch-1 and Notch-4 are all upregulated in rat pulmonary artery. Furthermore, an in vitro experiment also showed that the vessel wall thickness of cultured vascular strips from rats increases after hypoxia treatment, which can be decreased approximately 30% by *N*-(*N*-(3,5-difluorophenacetyl)-l-alanyl)-*S*-phenylglycine t-butyl ester (DAPT), a specific inhibitor of the γ-secretase [34]. These studies indicated to us that Notch signal in ECs is involved in neointimal thickness.

Notch signaling may have dual function in EC proliferation, which is depending on the relative level of p21 expression (Figure 2A). Notch-1 expression is increased in human pulmonary artery ECs after hypoxia treatment, which prompts EC proliferation via downregulation of p21 [35]. Low levels of p21 may activate cyclin D and induce cell proliferation, but p21 becomes an inhibitor at high levels [36]. Noseda and others demonstrated that when primary cultured ECs reach confluence, the activity of Notch signaling is augmented, while p21Cip1 is downregulated [24,36]. They also found that EC growth arrest is mediated by the repression of mitogen-activated protein kinase (MAPK)/PI3K (phosphatidylinositol 3 kinase) signaling and by p21Cip1, which prevents nuclear localization of cyclin D/cdk4 (cyclin-dependent kinase 4) required for Rb (retinoblastoma gene product) phosphorylation and S-phase entry [24].

Furthermore, EC behavior is at least partially dependent on reactive oxygen species (ROS) level downregulated by the Notch pathway. Blockade of Notch signaling can increase ROS in human umbilical vein endothelial cells (HUVECs), in contrast, suppression of ROS generation abolishes Notch blockade-induced HUVEC proliferation [37].

## 4. Endothelial Cell Apoptosis

Except for regulating cell proliferation and regeneration, Notch also affects EC apoptosis and survival, key cellular behaviors associated with vascular remodeling processes (Figure 2B). Vascular injury activates Notch signaling promptly, which further destroys the balance between EC proliferation and apoptosis, eventually influencing neointimal hyperplasia.

During the development of transplant arteriosclerosis (TA), EC apoptosis leads to neointimal hyperplasia in aortic allografts and allograft dysfunction. EC apoptosis induces the production of TGF-β1 in both apoptotic and neighboring viable cells, resulting in increased TGF-β1 in the culture media. In transgenic rat aorta transplantation models, inhibition of EC apoptosis in B-cell lymphoma (Bcl)-xL(+/+) knock-in rat aortic allografts significantly reduces TGF-β1 production in both allograft endothelia and blood plasma, which in turn decreases accumulation of SM22α+ cells from transgenic recipient ECs in neointima and alleviated TA [38].

It has been reported that the transcript levels of Notch-2, -3, and -4 are markedly downregulated in TA. In Quillard et al.’s research, TA correlates with high levels of tumor necrosis factor (TNF), TGF-β and interleukin (IL)-10. They also found that Notch-4 expression is decreased in transplants and cultured ECs. Further knockdown of Notch-4 and HES-1 by small interfering RNA (siRNA) promotes ECs apoptosis. As expected, silencing Notch-4 or HES-1 drastically inhibits repair of endothelial injury [39]. Mackenzie et al.’s studies demonstrated that Notch-4 provides endothelial protection in two ways: inhibition of the c-Jun N-terminal kinase (JNK)-dependent pro-apoptotic pathway in an RBP-Jκ-dependent manner and induction of an anti-apoptotic pathway through an RBP-Jκ-independent upregulation of Bcl-2 [40].

However, after Notch-2 ICD transduction in cultured human arterial endothelial cells (HAEC) and HUVECs or induced Notch-2 expression in HUVECs, EC apoptosis is promoted, notably through inhibiting the expression of survivin [41,42]. In addition, using a mouse model of pulmonary arterial hypertension, Li et al. showed that activation of nuclear factor kappa B (NF-κB) upregulates the expression levels of Notch-3, proapoptotic gene caspase 3 and Bax, downregulates antiapoptotic gene Bcl-2 expression in lung microvascular endothelial cells, which leads to EC apoptosis and endothelial-mesenchymal transition (EndMT) occurring in the lung [43]. Moreover, through activating Notch-1, HES-1 and caspase-3, accelerated cell apoptosis has been observed in human Eahy926 cells treated with high glucose [44].

All of this evidence supports that Notch activation is involved in neointimal formation through regulating EC apoptosis and survival. It is possible that the four different Notch receptors have different roles in EC apoptosis.

## 5. Endothelial-Mesenchymal Transition

EndMT is a specific form of epithelial-mesenchymal transition (EMT), which is an important biologic transdifferentiation process that participates in embryogenesis, organ development, tissue regeneration, organ fibrosis and cancer metastasis [45]. Similar to the process of EMT, when undergoing EndMT, ECs lose their endothelial specific markers, such as CD31, also known as platelet endothelial cell adhesion molecule-1 (PECAM-1) or VE-cadherin, gain mesenchymal markers, such as α-smooth muscle actin (α-SMA) or fibroblast-specific protein 1 (FSP-1, also known as S100A4), lose cell-cell junctions and acquire invasive and migratory properties [46,47].

EndMT has emerged as a player in the pathogenesis of vascular neointimal hyperplasia. Notintimal cells may arise through migration and proliferation of VSMCs; recent studies showed that the endothelium is also a source of smooth muscle-like cells [48]. It has been reported that EndMT occurs in myoendothelial cells in human atherosclerotic plaques and porcine aortic tissues. In vitro and in vivo experiments all showed that ECs exposed to disturbed flow undergo EndMT, which contributes to neointimal hyperplasia and induces atherogenic differentiation of ECs [49]. Through introducing endothelial-specific deletion of fibroblast growth factor receptor substrate 2 α (Frs2α) in atherosclerotic ApoE(−/−) mice, Chen et al. reported that these double-knockout mice exhibit extensive development of EndMT and increased neointimal formation. Furthermore, patients with coronary atherosclerosis showed that the loss of endothelial fibroblast growth factor receptor1 (FGFR1) expression leads to activation of endothelial TGF-β signaling and the development of EndMT in atherosclerotic plaques [50]. In vivo murine cell lineage-tracing models also presented that endothelial-derived cells contribute to neointimal formation through EndMT, which is dependent on the activation of the TGF-β mediated Smad2/3-Slug signaling pathway [51].

As a major regulator of cell phenotype, Notch is involved in the process of EndMT. Noseda et al. provided the first evidence that Jagged-1/Notch interactions induce endothelial-to-mesenchymal transformation. Notch activation in ECs results in morphological, phenotypic and functional changes, which is consistent with mesenchymal transformation [52]. Notch and TGF-β/smad3 signaling synergistically induce Snail expression in ECs and promote EndMT in cardiac cushion morphogenesis [53]. Blocking the Notch signaling pathway by using DAPT, EndMT in rat corneal ECs induced by TGF β1, -β2 or -β3 is prevented and the transformed ECs are reversed to a normal phenotype [54].

Neointimal hyperplasia occurs seriously in arteriovenous fistulas (AVFs) of chronic kidney disease (CKD) mice or patients. ECs of AVFs in CKD mice or patients express mesenchymal markers (FSP-1 and/or α-SMA) and exhibit increased expression and nuclear localization of the Notch intracellular domain. Uremic mice also show a decreased expression of VE-cadherin, whereas the expressions of Notch-1, -4, RBP-Jκ and Notch target genes are increased in ECs of AVFs. Blockade of the Notch pathway by DAPT or by RBP-Jκ knockout suppresses neointimal formation in mice [23]. Thus, data in the literature suggest that the Notch pathway is correlated with EndMT and contributes to the neointimal hyperplasia in vascular remodeling (Figure 2C).

## 6. The Contact Interaction between Endothelial Cells and Smooth Muscle Cells

Vessel wall is mainly composed of ECs and parietal cells (VSMCs and pericytes). The direct communication between ECs and VSMCs through myoendothelial gap junctions and microprojections has been widely known [55,56]. In addition, Notch ligand-receptor interaction is another direct communication means between ECs and VSMCs. Contact-mediated activation of Notch signaling plays important roles in cell and vessel maturation, survival and homeostasis. In these processes, different Notch receptors may have specific roles [57].

In vertebrates, ECs can express three Notch ligands (Dll-4, Jagged-1 and -2) and all of the known Notch receptors (Notch-1, -2, -3 and -4), while VSMCs express ligand Jagged-1 and three receptors (Notch-1, -2 and -3) [15]. We summarized the effects of ECs-VSMCs interaction mediated by different Notch receptor-ligand on neointimal formation in Table 1A. During vascular development, VSMCs recognize Notch ligand Jagged-1 on ECs and induce the expression of integrin αvβ3 in VSMCs, which facilitates VSMCs adhering to endothelial basement membrane and promotes vessel maturation [58]. In post-development vessels, the endothelial protein kinase B (PKB, also known as Akt) deletion reduces the expression of endothelial Jagged-1 and leads to the gradual loss of VSMCs due to diminished Jagged-1/Notch signaling. It sustains that contact-mediated activation of Notch signaling is critical in maintaining vascular stability and homeostasis [59]. Among the Notch signaling molecules, EC membrane ligand Jagged-1 is required for the induction of Notch-3 in VSMCs and inducing VSMC differentiation [60,61]. Neither the addition of soluble Jagged-1 nor EC-conditioned medium induces VSMC differentiation, while co-cultured ECs with VSMCs induce VSMC differentiation; furthermore, knockdown Jagged-1 expression in ECs can abrogate the co-cultured VSMC phenotype change. All of this evidence strongly supports that the direct heterocellular cell-cell contact is necessary for regulating VSMC differentiation via Jagged-1/Notch-3 signaling [62].

Except for Notch-3, Notch-2 is also activated in VSMCs co-cultured with ECs. Both Notch-2 and Notch-3 in VSMCs are mediators of EC-induced differentiated phenotype and contribute to increased contractile protein expression. However, the two receptors have separate and distinct functions. Notch-2 is specifically required for the suppression of cell proliferation, while Notch-3 is mainly responsible for VSMC secretory function [63].

VSMC proliferation, phenotype change and ECM secretion are hallmarks of neointimal formation in cardiovascular diseases and vascular surgery. Balloon injury induced Notch-1, Notch-3 and Jagged-1 expression in rat carotid arteries. However, soluble Jagged-1 inhibits neointimal formation after balloon injury or vein graft by decreasing VSMC proliferation and migration through interference with the Notch signaling pathway [64]. This evidence suggested that inhibition of neointimal formation may be due to inactivation of the Notch signaling pathway through soluble Jagged-1 competing with EC Jagged-1 to bind with the Notch receptor.

Reciprocally, VSMCs’ Jagged-1 activate Notch pathway in ECs through the Notch-1 receptor and induce EC proliferation. Notch signaling-deficient primary VSMCs have reduced proliferation and migration capacities and a diminished expression of Jagged-1 ligand. After being co-cultured with such VSMCs, ECs exhibit reduced growth rates and lower levels of activated Notch-1 receptor (Notch-1ICD) [65]. As discussed previously, EC proliferation and regeneration are critical events in neointimal formation. Through regulating the contact between ECs and VSMCs, activation of Notch could be manipulated, which may represent a unique therapeutic target to improve neointimal formation after vascular injury.

Notch signaling between cells can also be transmitted by exosomes from a distance. Dll-4 is incorporated into endothelial exosomes; the Dll-4 containing exosomes can freely travel through the 3D collagen matrix, transfer Dll-4 protein to distant tip cells and induce tip cell retraction [72]. However, whether there exists exosome-mediated Notch signaling between ECs and VSMCs in neointimal formation has not yet been reported.

**Figure 3:**
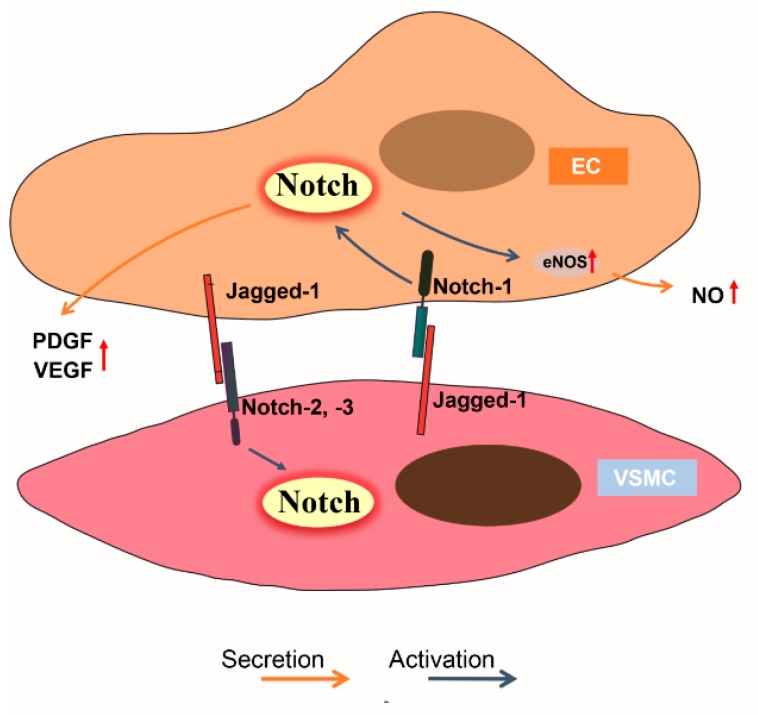
Notch signaling in cell-cell communication between endothelial cells (ECs) and vascular smooth muscle cells (VSMCs). In the process of direct cell-cell interaction between ECs and VSMCs, EC membrane ligand Jagged-1 is recognized by Notch-2, -3 in VSMCs; reciprocally, VSMCs Jagged-1 binds with EC Notch-1 receptor and activates the Notch downstream pathway in ECs (also see Table 1A). In addition, activation of Notch signaling induces nitric oxide (NO), platelet-derived growth factor (PDGF), vascular endothelial growth factor (VEGF) and other factors’ production from ECs via autocrine and paracrine process; these factors can regulate EC and VSMC proliferation, migration, differentiation and play different roles in neointimal hyperplasia (see Table 1B).

Taken together, Jagged-1 seems to be the only ligand in the Notch pathway that plays an important role in both ECs and VSMCs during neointimal formation development. Notch-1 may be the only receptor in the EC lineage that recognizes the Notch ligand in VSMCs, whereas several receptors (Notch-2, -3) are involved in the VSMC lineage to recognize the Notch ligand in ECs (Figure 3).

## 7. Endothelial Cell Secretory Function

Although the vascular endothelium is made up from only a single layer of ECs, it works as an “endocrine organ” via an autocrine and/or paracrine process and contributes to vascular homeostasis, such as angiogenesis, inflammation, platelet aggregation and vascular remodeling [73,74]. The regulatory molecules derived from ECs include non-growth factors and growth factors, such as nitric oxide (NO), VEGF, platelet-derived growth factor (PDGF), basic fibroblast growth factor (bFGF) and insulin-like growth factor-1, etc. [75,76] (Figure 3). There are many reports about crosstalk between Notch signaling and the regulatory molecules mentioned above; we refer the readers to the three main kinds of factors derived from ECs regulated by Notch signaling and their effects on neointimal hyperplasia (Table 1B).

Mainly produced by endothelial NO synthase (eNOS), NO derived from EC is an important mediator of normal and pathologic vascular remodeling [77]. NO not only confers anti-platelet and anti-inflammatory properties to the vessels, but also promotes EC proliferation [66], inhibits VSMC proliferation and migration [68], controls M1 macrophage polarization [78], regulates redox balance [79], keeps the stability and function of blood vessels and suppresses neointimal hyperplasia [80]. A recent study reported that Notch induces Activin A expression, thereby activating the PI3K/Akt pathway to phosphorylate eNOS and promoting NO production [67]. Inhibition of Notch decreases endothelial NO production by reduced eNOS expression [81]. In vein grafts of aged rats, the reduced expression of Dll-4 and Notch-4 has been found, which is associated with the decreased eNOS protein expression, reduced eNOS membrane targeting and colocalization with caveolin-1, as well as significantly thicker neointima [82].

The contribution of VEGF in neointimal formation has been widely evaluated. Recent studies demonstrated that VEGF can block neointimal formation through inducing EC growth and reducing VSMC growth after vascular injury [83]. In addition, bone marrow-derived mesenchymal stem cells treated with VEGF differentiate into endothelial-like cells and significantly attenuate neointimal thickness [84]. However, lentivirus-mediated VEGF-A inhibition can decrease the venous neointimal hyperplasia of AVFs [85]. This evidence suggested that the effect of VEGF on neointimal formation is complicated. Although there is no direct evidence to support that Notch signaling mediates neointimal hyperplasia through autocrine or paracrine of VEGF in ECs, inhibition of Notch-1 or Notch-4 can block thymosin β 4-induced VEGF expression in HUVECs [86]. In contrast, many studies uncovered that VEGF can activate Notch signaling in ECs and act as an upstream mediator of the Notch pathway [87,88].

As a smooth muscle cell growth and survival factor, PDGF also plays a prominent role in VSMCs migrating into the neointima following acute injury or in atherosclerosis. High shear stress inhibits arterial wall thickening in vivo, which may be related to enhanced activation of PDGF-R alpha in VSMCs by PDGF isoforms secreted from the endothelium. The neutralizing antibody against PDGF-AA enhances VSMC migration; in contrast, antibodies against PDGF-BB abolish VSMC migration [70]. VSMC-rich neointimal formation is accelerated in the ligated carotid artery of mice treated with erythropoietin delta, by which the expression and release of PDGF-B is induced in HUVECs [69]. Findings also showed that PDGF-BB and PDGF-DD are all VSMC phenotypic modulators. PDGF-DD expression is increased in neointimal lesions in the aortic arch region of apolipoprotein C-deficient ApoE(−/−) mice. In addition, human ECs exposed to an atherosclerosis-prone flow pattern, as in vascular regions susceptible to the development of atherosclerosis, exhibit a significant increase in PDGF-DD expression [71].

Several recent studies reported that Notch signaling is involved in regulating PDGF production from ECs. Exposure of human brain microvascular endothelial cells to DAPT or silencing of Notch-1 results in abrogation of cocaine-mediated induction of PDGF-B. The study provided the first evidence of the involvement of Notch-1 activation in PDGF-B expression [89]. β-catenin, a key signal molecule of the Wnt/β-catenin-Dll4/Notch signaling cascade in endothelia, its transcriptional activity directly regulates the endothelial expression of PDGF-B [90]. Conditional medium from matricellular protein, secreted protein acidic and rich in cysteine (SPARC) overexpressed neuroblastoma cells show suppressed expression of VEGF, PDGF, FGF and matrix metalloprotein 9 in ECs, which is mediated by the inhibition of the Notch signaling pathway [91]. Taken together, these findings suggest that PDGF secretion induced by Notch may be involved in neointimal hyperplasia.

There are also many other EC-derived molecules that have been proven or speculated to influence neointimal hyperplasia. For instance, apoptotic ECs actively release paracrine mediators’ C-terminal fragment of perlecan and epidermal growth factor, which inhibit apoptosis of mesenchymal stem cells (MSC), which are pivotal to vascular repair and neointimal formation [92]. As a secreted glycoprotein that has been implicated in regulating VSMC proliferation and migration, the downregulated expression of apolipoprotein D (APOD) is partly caused by paracrine secretion of ECs. In addition, Notch-3 on mural cells also promotes the downregulation of APOD, possibly through interaction with the Jagged-1 ligands on ECs [93].

## 8. Endothelial Cell Barrier Dysfunction

The development of functional blood vessel requires an integrated layer of endothelial cells. Based on the EC junction, the structural and functional integrity of the endothelium is fundamental for maintaining vascular homeostasis [94]. Destruction of the protective endothelial barrier will subsequently lead to vascular injury and neointimal formation. Changes in shear and/or hoop stress, direct drug-induced cytotoxicity, mechanical device implant-induced injury or inflammatory response in vascular surgery or cardiovascular surgery induce vascular injury and cause EC dysfunction [95]. As discussed above, vascular injury is a complex cascade of events involving endothelial denudation, the release of growth factors and cytokines which triggers platelet degranulation and aggregation, subsequent inflammatory cells or mediators invading the injured locations, smooth muscle cell proliferation and migration to form neointimal hyperplasia at the subendothelial space. Reciprocally, platelet activation and inflammation response lead to delayed re-endothelialization and endothelial dysfunction [96].

**Figure 4:**
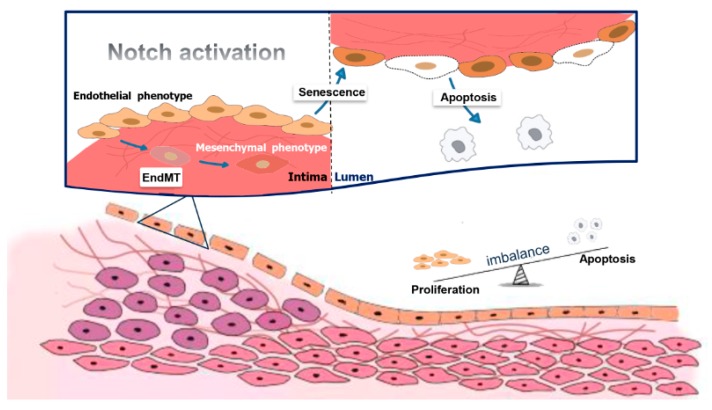
Endothelial cell (EC) dysfunction caused by Notch signaling leads to neointimal hyperplasia. When vascular injury occurs, Notch signaling is activated, which further triggers EC phenotype change and causes EC dysfunction. In these processes, ECs acquire the senescent phenotype, the apoptotic phenotype or the mesenchymal phenotype and lose their barrier function, which leads to endothelial hyperpermeability, leakage and inflammatory responses. Furthermore, vascular smooth muscle cell -like cells can transmigrate into the media and proliferate, resulting in neointimal hyperplasia.

Endothelial dysfunction is characterized by EC phenotype change with impaired endothelium-dependent barrier and imbalance between re-endothelialization and apoptosis or growth inhibiting and growth-promoting substances. Notch signaling cascades are involved in and partially responsible for EC dysfunction events (Figure 4). For example, apoptotic or senescent phenotype ECs lose their barrier function. Activation of Notch increases myosin light chain phosphorylation by activating Rho kinase, which further triggers EC acquiring senescence phenotype and leads to hyperpermeability of the endothelium [97]. In atherosclerosis, Notch activation induces EC senescence and prompts the expression and secretion of pro-inflammatory cytokines such as IL-6, IL-8, IL-1α. Among these factors, the upregulated IL-6 may mediate leukocyte transendothelial migration [98]. In addition, Notch activation also causes EC to acquire VSMC phenotype, which leads to ECs losing their barrier function and induces neointimal hyperplasia [23]. Consequently, attenuation of Notch signaling in ECs might provide a treatment strategy for neointimal formation.

As mentioned above, activation of Notch-2 induces EC apoptosis while Notch-4 plays a protective role [39,43]. In arterial ECs, pro-inflammatory cytokine TNF-α elicits a switch in Notch expression, which is characterized by Notch-2 predominance over Notch-4. The events lead to a reduced Notch activity, then promoting caspase-dependent EC apoptosis and vascular dysfunction [99]. In most cases, the upregulated expressions of Jagged-1, Dll-4, Notch-1 and Notch-4 are associated with inhibition of EC proliferation and vascular dysfunction [100,101]. This evidence proved that loss of ECs could lead to increased platelet reactivity and VSMC proliferation, thereby facilitating neointimal formation.

EC dysfunction may also be related to the change of the secretory function of ECs. Among the substances released from ECs, NO is involved in the impairment of endothelium-dependent vasodilatation. Endovascular interventions are associated with diminished bioavailability of NO and increased local inflammatory response, which triggers EC apoptosis [102,103,104]. Reducing eNOS expression or promoting inducible nitric oxide synthase (iNOS) expression induces EC apoptosis and dysfunction, which can be mediated through the Jagged-1/Notch pathway [105]. To conclude, enhanced degradation of NO and decreased eNOS expression and/or activity leads to EC dysfunction and contributes to neointimal formation. As shown in Figure 4, we summarized the relationship between Notch signaling and EC dysfunction during neointimal formation development.

## 9. Limits and Perspectives

We have reviewed here the accumulated evidence that the Notch pathway is involved in multiple aspects of EC key functions (proliferation, regeneration, apoptosis, differentiation, cell-cell interaction) and contributes to neointimal formation. However, our review also found a number of apparent inconsistencies and problems in these studies. Although the relative level of p21 expression may decide EC growth after Notch activation, no data show the exact expression level or range of p21. The patterns of Notch-1 to -4 expression can play different roles in EC apoptosis, but which components function at what apoptosis steps in ECs? Notch signaling seems to induce endothelial mesenchymal phenotype switch, so does there exist a specific relationship among ligands, receptors, and target genes in the process of transdifferentiation? How does Notch play a role in EC regeneration and differentiation? Except NO, how does the Notch pathway play any role in the regulation of other cytokines secreted from ECs to cause EC dysfunction? How about the crosstalk between Notch and other signaling pathways? Given that the Notch has a significant role in vascular development, further understanding of the Notch signaling pathway in the context of vascular biology will likely provide novel insights into the mechanisms of neointimal formation and new opportunities for rational therapeutic intervention.

## Figures and Tables

**Table 1:** The Notch pathway-mediated interaction between endothelial cells (ECs) and vascular smooth muscle cells (VSMCs).

**A. The Direct Contact between ECs and VSMCs Mediated by Notch**				
**Ways of Interaction**	Ligand	Receptor	Function	Possible Effect on Neointimal Hyperplasia
**Direct Contact**	ND	VSMC Notch-1	VSMC migration [64]↑	Promotion
	EC Jagged-1	VSMC Notch-2	VSMC differentiation [63]↑	ND
			VSMC proliferation [63]↓	
	EC Jagged-1	VSMC Notch-3	VSMC differentiation [60,61,62,63]↑	Promotion
			VSMC secretion [63]↑	
			VSMC migration [64]↑	
	VSMC Jagged-1	EC Notch-1	EC proliferation [65]↑	Inhibition
	ND	EC Notch-2, -3, -4 [15]	ND	ND
**B. The Indirect Communication between ECs and VSMCs Mediated by Notch Activation**				
**Ways of Interaction**	Notch Signaling	Molecules Secreted from EC	Function	Possible Effect on Neointimal Hyperplasia
**Indirect Communication**	Activation	NO	EC proliferation [66,67]↑	Inhibition
			VSMC proliferation [68]↓	
			VSMC migration [68]↓	
		VEGF	EC proliferation↑	Inhibition
			EC regeneration↑	
			VSMC proliferation↓	
		PDGF	VSMC proliferation [69]↑	Promotion
			VSMC migration [70]↑	
			VSMC differentiation [71]↑	

ND indicates not described; NO: nitric oxide; PDGF: platelet-derived growth factor; VEGF: vascular endothelial growth factor; ↑ indicates promotion; ↓ indicates inhibition.

## Abbreviations

ADAM	a disintegrin and metalloproteinases
AGS	alagille syndrome
APOD	apolipoprotein D
AVF	arteriovenous fistula
AVG	arteriovenous graft
bFGF	basic fibroblast growth factor
CACs	circulating angiogenic cells
CADASIL	cerebral autosomal-dominant arteriopathy with subcortical infarcts and leukoencephalopathy
CKD	chronic kidney disease
CVD	cardiovascular diseases
DAPT	*N*-[*N*-(3,5-Difluorophenacetyl)-l-alanyl]-*S*-phenylglycine t-butyl ester
Dll	delta-like
EC	endothelial cell
ECM	extracellular matrix
EMT	Epithelial-mesenchymal transition
EndMT	Endothelial-mesenchymal transition
eNOS	endothelial NO synthase
EPC	endothelial progenitor cell
FGFR1	Fibroblast growth factor receptor1
Frs2α	Fibroblast growth factor receptor substrate 2 α
FSP-1	fibroblast-specific protein 1
HAEC	human arterial endothelial cell
HERP	HES-related repressor protein
HES	hairy and enhancer of split
HUVEC	human umbilical vein endothelial cell
IFNγ	Interferon γ
IL	interleukin
iNOS	inducible nitric oxide synthase
MAML	Mastermind-like
MAPK	mitogen-activated protein kinase
MSC	mesenchymal stem cell
NF-κB	nuclear factor κB
NICD	Notch intracellular domain
NO	Nitric oxide
PDGF	platelet-derived growth factor
PECAM-1	platelet endothelial cell adhesion molecule-1
Rb	retinoblastoma
ROS	reactive oxygen species
α-SMA	α-smooth muscle actin
SPARC	secreted protein acidic and rich in cysteine
TA	transplant arteriosclerosis
TGF-β	transforming growth factor-β
TNF	tumor necrosis factor
VEGF	vascular endothelial growth factor
VSMC	vascular smooth muscle cell
WT	wild type
